# Supplementary material for: Comparative Analysis of Perceived Threat Threshold from Various Drivers to Cranes Along Indus Flyway, Punjab, Pakistan
Source: Biology (Basel). 2025 Sep 16;14(9):1275. doi: 10.3390/biology14091275 (PMC12467742; doi:10.3390/biology14091275)
Supplement: Supplementary file 1 [file biology-14-01275-s001.zip › Table S1.pdf]

Table S1. Results of Kruskal-Wallis post-hoc comparisons (Dunn's test with Bonferroni adjustment) showing significant regional differences in threat levels to cranes, particularly involving Khushab and Rajanpur.

Dunn (1964) Kruskal-Wallis multiple comparison p-values adjusted with the Bonferroni method.			
**Comparison**	**Z**	**P.unadj**	**P.adj**
Attock-Bhakkar	2.43313371	1.496877e-02	4.191256e-01
Attock-DG Khan	0.86686545	3.860157e-01	1.000000e+00
Bhakkar-DG Khan	-1.56626826	1.172858e-01	1.000000e+00
Khushab-Attock	5.08298378	3.715514e-07	1.040344e-05
Bhakkar-Khushab	2.64985007	8.052750e-03	2.254770e-01
Khushab-DG Khan	4.21611833	2.485434e-05	6.959215e-04
Attock-Layyah	1.88258659	5.975642e-02	1.000000e+00
Bhakkar-Layyah	-0.55054712	5.819442e-01	1.000000e+00
DG Khan-Layyah	1.01572113	3.097622e-01	1.000000e+00
khushab-Layyah	-3.2003972	1.372383e-03	3.842673e-02
Attock-Mianwali	0.04925372	9.607171e-01	1.000000e+00
Bhakkar-Mianwali	-2.38387999	1.713119e-02	4.796733e-01
DG Khan-Mianwali	-0.81761173	4.135789e-01	1.000000e+00
Khushab-Mianwali	-5.03373006	4.810269e-07	1.346875e-05
Mianwali-Layyah	-1.83333287	6.675308e-02	1.000000e+00
Attock-Muzaffargarh	0.93910424	3.476772e-01	1.000000e+00
Bhakkar-Muzaffargarh	-1.49402947	1.351679e-01	1.000000e+00
Muzaffargarh-DG Khan	0.07223879	9.424119e-01	1.000000e+00
Muzaffargarh-khushab	-4.14387954	3.414792e-05	9.561417e-04
Muzaffargarh-Layyah	-0.94348235	3.454342e-01	1.000000e+00
Muzaffargarh-Mianwali	0.88985052	3.735462e-01	1.000000e+00
Attock-Rajanpur	-1.15800966	2.468601e-01	1.000000e+00
Bhakkar-Rajanpur	-3.59114337	3.292305e-04	9.218453e-03
DG Khan-Rajanpur	-2.02487511	4.288019e-02	1.000000e+00
Khushab-Rajanpur	-6.24099344	4.348005e-10	1.217441e-08
Layyah-Rajanpur	-3.04059624	2.361102e-03	6.611086e-02
Mianwali-Rajanpur	-1.20726337	2.273307e-01	1.000000e+00
Muzaffargarh-Rajanpur	-2.09711389	3.598349e-02	1.000000e+00
Attock-Bhakkar	2.40520463	1.616341e-02	0.452575547
Attock-DG khan	0.80729476	4.194967e-01	1
Bhakkar-DG Khan	-1.59790987	1.100630e-01	1
Attock-Khushab	3.09073798	1.996597e-03	0.055904725
Bhakkar-Khushab	0.68553336	4.930074e-01	1
Khushab-DG Khan	2.28344323	2.240427e-02	0.627319655
Attock-Layyah	1.78972577	7.349801e-02	1

Bhakkar-Layyah	-0.61547885	5.382385e-01	1
DG Khan-Layyah	0.98243102	3.258876e-01	1
Khushab-Layyah	-1.30101221	1.932543e-01	1
Attock-Mianwali	1.39719816	1.623540e-01	1
Mianwali-Bhakkar	-1.00800647	3.134514e-01	1
DG Khan-Mianwali	0.5899034	5.552554e-01	1
Khushab-Mianwali	-1.69353983	9.035276e-02	1
Mianwali-Layyah	-0.39252762	6.946684e-01	1
Attock-Muzaffargarh	0.84899387	3.958847e-01	1
Bhakkar-Muzaffargarh	-1.55621076	1.196580e-01	1
DG Khan-Muzaffargarh	0.04169911	9.667386e-01	1
Khushab-Muzaffargarh	-2.24174412	2.497791e-02	0.699381612
Muzaffargarh-Layyah	-0.94073191	3.468423e-01	1
Muzaffargarh-Mianwali	-0.54820429	5.835516e-01	1
Attock-Rajanpur	-1.04748163	2.948775e-01	1
Bhakkar-Rajanpur	-3.45268625	5.550342e-04	0.015540956
DG Khan-Rajanpur	-1.85477638	6.362817e-02	1
Khushab-Rajanpur	-4.13821961	3.500113e-05	0.000980032
Layyah-Rajanpur	-2.8372074	4.551004e-03	0.127428109
Mianwali-Rajanpur	-2.44467978	1.449808e-02	0.405946244
Muzaffargarh-Rajanpur	-1.89647549	5.789720e-02	1
