# Supplementary material for: Comparative Analysis of Perceived Threat Threshold from Various Drivers to Cranes Along Indus Flyway, Punjab, Pakistan
Source: Biology (Basel). 2025 Sep 16;14(9):1275. doi: 10.3390/biology14091275 (PMC12467742; doi:10.3390/biology14091275)
Supplement: Supplementary file 1 [file biology-14-01275-s001.zip › Translated Questionaire (File S1).pdf]

Questionnaire for Assessing Threats to Migratory Cranes in Punjab, Pakistan

Section A: Respondent Information

1. **Respondent ID (Optional)**: __________
2. **Name (optional)**: __________________________
3. **Gender**:
   ☐ Male ☐ Female ☐ Other
4. **Age**: ______ years
5. **Occupation**:
   ☐ Farmer ☐ Fisherman ☐ Shepherd ☐ Wildlife Official ☐ Student ☐ Hunter ☐ Electrical Technician ☐ Stock Market Official ☐ Other: ____________
6. **Education Level**:
   ☐ No Formal Education (Level 1)
   ☐ Primary Education (Level 2)
   ☐ Secondary Education (Level 3)
   ☐ Higher Secondary Education (Level 4)
   ☐ University or Equivalent (Level 5)

Section B: Threat Location and Context

7. **Region / District**: ___________________
8. **Location of Threat**:
   ☐ Riverbank ☐ Barren Land ☐ Agricultural Land ☐ Forest Area ☐ Other: ________
9. **Crane Species Most Often Targeted**:
   ☐ *Grus grus* (Eurasian Crane)
   ☐ *Grus virgo* (Demoiselle Crane)
   ☐ Both

Section C: Type of Threat and Impact

10. **What type of threat have you observed/Heard?**
   ☐ Illegal Killing / Hunting
   ☐ Domestication (Capturing for Pets/Farms)
   ☐ Trading / Trafficking
   ☐ Utility Collision (e.g., power lines)

☐ Predation (e.g., jackals, dogs)
☐ Other: ___________

11. **What methods are used for this threat?**
☐ Shooting
☐ Live Capturing
☐ Power Line Collision
☐ Attack by Predators
☐ Other: ___________

12. **How often does this threat occur in your area? (Frequency)**
Rate from 1 (Very Rare) to 5 (Very Frequent): ☐1 ☐2 ☐3 ☐4 ☐5

13. **How serious is the impact of this threat on cranes? (Severity)**
Rate from 1 (Not Severe) to 5 (Extremely Severe): ☐1 ☐2 ☐3 ☐4 ☐5

14. **Are there hunting camps or gatherings in your area?**
☐ Yes ☐ No

- If yes, please mention camp name/ID (if known): __________
- Estimated number of hunting parties: ____________

Section D: Electrocution and Predation

15. **Have you seen cranes die due to electrocution or power line collision?**
☐ Yes ☐ No

- If yes, how often? (1–5 scale): __________
- Where did this happen? ___________________

16. **Are cranes being preyed on by animals (e.g., jackals, dogs)?**
☐ Yes ☐ No

- If yes, how often? (1–5 scale): __________
- Which predators are involved? ______________

Section E: Cultural and Motivational Aspects

17. **Is crane hunting/trapping part of a cultural or traditional practice in your area?**
☐ Yes ☐ No

- If yes, please describe briefly: _______________________________

18. **What are the common reasons for hunting or capturing cranes?**
☐ Food ☐ Trade ☐ Pets ☐ Tradition ☐ Other: ___________

Section F: Ethics and Consent

19. **Did the researcher explain the purpose of this study to you?**
☐ Yes ☐ No

20. **Were you informed that your answers will be confidential and you can withdraw at any time?**
☐ Yes ☐ No

Note:

- **Severity*** The perceived level of harm caused by the threat to crane populations on a scale of 1 (minimal) to 5 (extreme).
- **Frequency*** The respondent's observation of how often a particular threat occurs, on a scale of 1 (rarely) to 5 (very frequently).
